# Are exercises with or without occlusal splints more effective in the reduction of pain in patients with temporomandibular disorders of myogenic origin? A systematic review

**DOI:** 10.1590/1678-7757-2022-0298

**Published:** 2023-01-23

**Authors:** Jessica Fernanda de Oliveira Lima BATISTA, Taciana Emília Leite VILA-NOVA, Sandra Lúcia Dantas MORAES, Eduardo Piza PELLIZZER, Belmiro Cavalcanti do Egito VASCONCELOS, Jéssica Marcela de Luna GOMES, Cleidiel Aparecido Araújo LEMOS, Mônica Vilela HEIMER

**Affiliations:** 1 Universidade de Pernambuco Faculdade de Odontologia Recife PE Brasil Universidade de Pernambuco (UPE), Faculdade de Odontologia, Santo Amaro, Recife, PE, Brasil.; 2 Universidade de Pernambuco Faculdade de Odontologia Departamento de Reabilitação Oral Recife PE Brasil Universidade de Pernambuco (UPE), Faculdade de Odontologia, Departamento de Reabilitação Oral, Santo Amaro, Recife, PE, Brasil.; 3 Universidade Estadual Paulista Faculdade de Odontologia de Araçatuba Departamento de Materiais Dentários e Prótese Dentária Araçatuba SP Brasil Universidade Estadual Paulista (UNESP), Faculdade de Odontologia de Araçatuba, Departamento de Materiais Dentários e Prótese Dentária, Araçatuba, SP, Brasil.; 4 Universidade de Pernambuco Faculdade de Odontologia da Recife Departamento de Cirurgia Bucomaxilofacial da Pernambuco Recife PE Brasil Universidade de Pernambuco (UPE), Faculdade de Odontologia da Recife, Departamento de Cirurgia Bucomaxilofacial da Pernambuco, Santo Amaro, Recife, PE, Brasil.; 5 Centro Universitário FACOL-UNIFACOL Vitória de Santo Antão PE Brasil Centro Universitário FACOL-UNIFACOL, Vitória de Santo Antão, PE, Brasil.; 6 Universidade Federal de Juiz de Fora Departamento de Odontologia Campus Governador Valadares Governador Valadares MG Brasil Universidade Federal de Juiz de Fora, Departamento de Odontologia, Campus Governador Valadares, Governador Valadares, MG, Brasil.; 7 Universidade de Pernambuco Faculdade de Odontologia Departamento de Odontopediatria Recife PE Brasil Universidade de Pernambuco (UPE), Faculdade de Odontologia, Departamento de Odontopediatria, Santo Amaro, Recife, PE, Brasil.

**Keywords:** Temporomandibular joint dysfunction syndrome, Occlusal splints, Physical therapy

## Abstract

Temporomandibular disorders (TMD) is a term used to describe a set of clinical conditions that may compromise the temporomandibular joint (TMJ) and masticatory muscles and/or associated structures, considered the most frequent cause of orofacial pain of non-dental origin. In recent years, many forms of physical therapy have been used in the treatment of TMD to reduce pain and improve the range of mandibular movement present in this impairment. Among these resources are kinesiotherapy (exercise), electrothermal and manual therapy, acupuncture, training posture, mobilizations, and biofeedback.

**Objectives:**

To determine if exercises with or without occlusal splints are effective in reducing pain in patients with temporomandibular disorders (TMD) of myogenic origin.

**Methodology:**

This systematic review was registered in the International Prospective Register of Systematic Reviews (CRD 42019134244). Controlled trials published in PubMed, Scopus, and Cochrane Library following PRISMA guidelines up to April 2022 were randomized and included. The population above 18 years, which evaluated the effectiveness of exercise with or without occlusal splints in reducing pain in patients with TMD of myogenic origin, diagnosed through the Research Diagnostic Criteria for Temporomandibular Disorders, was also included. There was no restriction on the period of publication. Cochrane risk of bias analysis was performed.

**Results:**

Of the five included articles, all showed a reduction of pain, but without significant differences between the interventions performed. Additionally, studies that evaluated the quality of life and mandibular movements showed a reduction in pain, but no significant differences between therapies.

**Conclusion:**

The analyzed studies showed no difference in the improvement of pain, quality of life, and mandibular movements between the groups that performed only exercises or the associated treatments.

## Introduction

Temporomandibular disorders (TMD) is a term used to describe a set of clinical conditions that may compromise the temporomandibular joint (TMJ) and masticatory muscles and/or associated structures,^1-3^ considered the most frequent cause of orofacial pain of non-dental origin.^3,4^ The signs and symptoms of TMD are sensitivity to palpation of the masticatory muscles, pain, restrictions of joint movement, noise in the temporomandibular joint (TMJ) headache, and tinnitus, among others.^4,6-9^

While TMD is uncommon in childhood, its prevalence increases in adolescence and young adulthood, affecting people mainly in the age range of 20 to 40 years. Females are the most affected, presenting greater symptom severity and experiencing more difficult recoverying.^7,8^ Its etiology is multifactorial, involving genetic, individual, and environmental factors. Studies showed that increased stress is associated with the appearance of muscular tension, which affects the functioning of the stomatognathic system and TMJ.^8,10^

TMD is a multifactorial pathogenesis, and its treatment must be interdisciplinary, combining a conservative approach, cognitive behavioral therapy, physical therapy, and pharmacology intraoral devices where applicable.^11^Occlusal splints (OS), which promotes the relaxation of the masticatory muscles, has been widely used aiming to restore neuromuscular balance. Moreover, it repositions the mandibular condyles and normalizes the proprioception of the periodontal ligament, providing a correct alignment of the temporomandibular joint. Such removable device is made of acrylic resin with polymer and can be used during the day or at night.^3,5,13-16^

In recent years, many forms of physical therapy have been used in the treatment of TMD to reduce pain and improve the range of mandibular movement present in this impairment. Although the literature lacks consensus on the best treatment for this disorder, some therapies such as kinesiotherapy (exercise), electrothermal and manual therapy, acupuncture, training posture, mobilizations, and biofeedback can be used in the management of TMD.^6,7,11,17^

Orofacial exercises have been one of the main interventions in the management of TMD. Aiming to promote the proper functioning of the cranio-cervico-mandibular region, strength, mobility, coordination, the relaxation of the jaw muscles, the increase of range of motion of the TMJ, proprioception, and muscle relaxation, this intervention brings positive effects on pain. The exercises must be part of an individualized program, in addition to the need for verbal and written instructions.^18,19,20^

This study aimed to perform a systematic review of the literature to verify if exercises with or without the OS are effective in the reduction of pain in patients with TMD of myogenic origin. The hypothesis of the study is the physical therapy exercises associated with the occlusal splint are more effective in the treatment of pain in myofascial TMD.

## Methodology

### Protocol and registration

This systematic review was conducted based on studies published in PubMed, Scopus, and Cochrane Library that followed PRISMA (Preferred Reporting Items for Systematic Reviews and Meta-Analyzes) guidelines^21^and were registered in PROSPERO under protocol No. CRD 42019134244.

### Eligibility criteria

The problem, intervention/indicator, comparison, and outcome of interest (PICO) question guiding this study was the following: *“Are exercises with or without the OS effective in reducing pain in patients with TMD of myogenic origin?”* Here, “P” was “patients with TMD of myogenic origin”; “I” was “physical therapy exercises”; “C” was “use of the OS + physical therapy exercises”; and “O” was “pain assessment (primary) and quality of life and mandibular movements (secondary).”

### Inclusion criteria

Randomized clinical trials published up to April 2022 with a population above 18 years, which evaluated the effect of exercise with or without occlusal splints (made of acrylic) in the reduction of pain in patients with TMD of myogenic origin, diagnosed using the Research Diagnostic Criteria for Temporomandibular Disorders (RDC/TMD), were included. There was no restriction on the period of publication.

### Exclusion criteria

Studies that used other tools for TMD diagnosis, assessed TMD unrelated to muscle origin, or included patients with diseases that could affect masticatory muscles or the TMJ, were excluded.

### Search strategy

**Figure 1:**
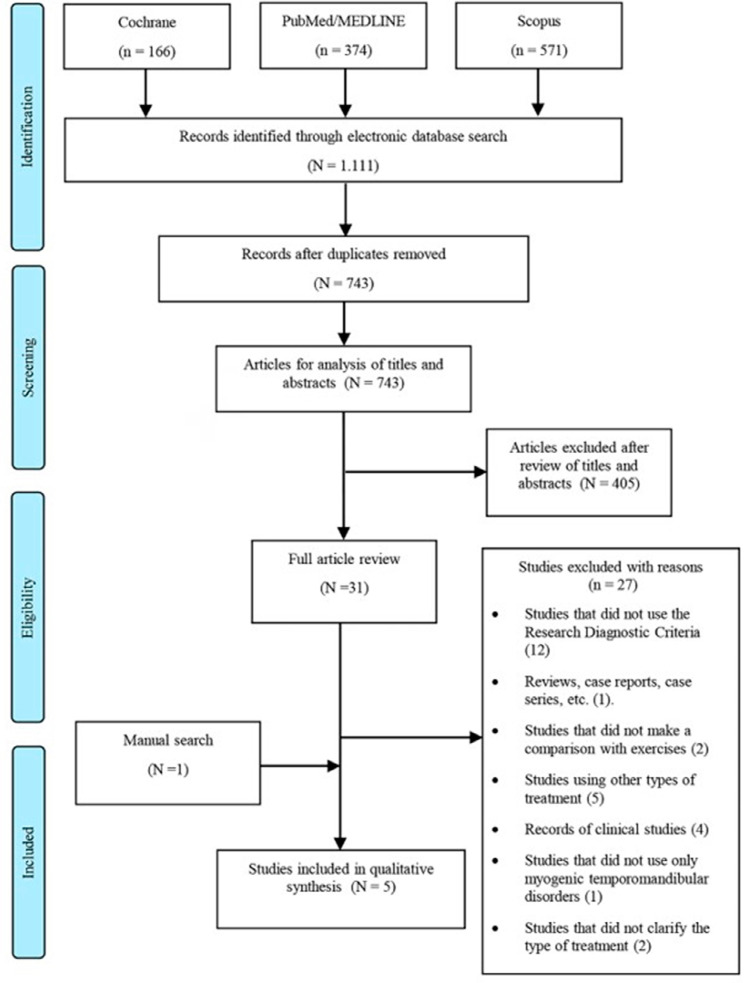
PRISMA flow diagram of study selection

Two authors (JFOLB and JMLG) independently performed the electronic search in the databases PubMed/MEDLINE, Cochrane Library, and SCOPUS. The keywords and MeSH descriptors were combined with Boolean operators for each database, as in Figure 1. A manual search was performed in the references of the articles included and in journals in the field of pain, rehabilitation, and temporomandibular dysfunction: “Head & Face Medicine,” “The Journal of Pain,” and “Journal of Oral Rehabilitation.”

### Study selection

The selection process was conducted in two phases. In the first phase, two independent evaluators (JFOLB and JMLG) read the titles and summaries of the studies identified in the databases searched. Studies that did not meet the inclusion criteria were discarded. In the second phase, the same researchers applied the eligibility criteria to the full text of the articles. During the searches, disagreements were resolved by a third reviewer (MVH).

Inter-examiner evaluation (kappa test) was performed to verify the level of agreement between the authors during the search and selection of the articles.

### Data collection

One author (JFOLB) collected the relevant data from the articles, which were then verified by two other authors (JMLG and MVH). The qualitative data collected included author/year/country, study design, follow-up, age group, intervention (types of exercises, habit counseling, and occlusal splints), assessment instrument for pain outcome, results (pain, quality of life, and mandibular movements), conclusion, and effect.

### Risk of bias

The bias risk analysis was performed by two independent authors (BCEV and SLDM) using the Cochrane risk of bias tool. For each manuscript, the items’ allocation confidentiality, the possibility of randomization, blinding, and patient loss were examined. Finally, the studies were categorized according to the level of risk of bias as “low,” “medium,” or “high”.

## Results

### Selection of studies

**Figure 2:**
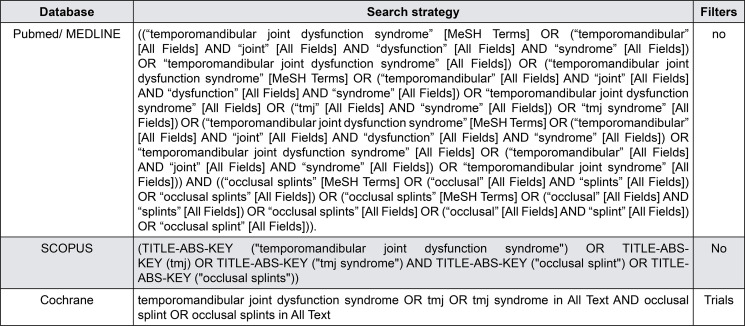
Search strategy for each database

The database search yielded a total of 1.111 studies in the databases as follows: 374 Pubmed/MEDLINE, 166 Cochrane Library, and 571 SCOPUS. After the exclusion of duplicated studies (368), 743 studies were evaluated through the reading of titles and abstracts. Among the 31 articles remaining for full-text evaluation, after applying the eligibility criteria, 27 were excluded for several reasons. Manually searching, we found one article that met the inclusion criteria. Finally, five articles were part of the data extraction and qualitative synthesis. Figure 2 shows the process of identification, inclusion, and exclusion of studies.

The inter-examiner test (kappa) showed “moderate” to “almost perfect” agreement according to the criteria (K=0.88) for each database.

### Characteristics of the studies

**Figure 3:**
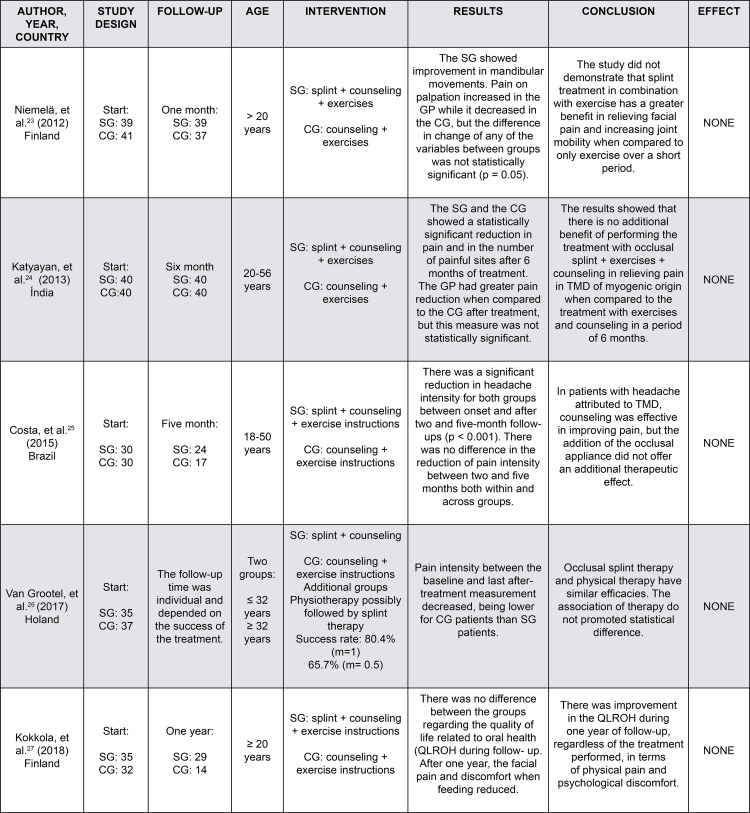
Characteristics of included studies

Figure 3 summarizes the articles, highlighting the authors, year of publication, country of origin, age group, sample, follow-up, intervention, assessment instrument for pain outcome, results, and conclusion. As for the temporal distribution, studies were conducted in 2012,^22^ 2013,^21^2015,^22^2017,^27^ and 2018.^24^Regarding geographic distribution, one study was conducted in Brazil, while the others were conducted in Finland,^20,24^ the Netherlands,^27^ and India.^21^The age range of the study populations was broad, covering both young and adults.^20,21,22,24,27^

The follow-up period varied among the studies: one month,^20^ five months,^22^six months,^21^and one year.^24^ In one study,^27^the follow-up time, dependent on the rate of symptom improvement, differed for each patient. Regarding the treatments performed, four studies associated exercise with occlusal splints,^20-22,24^and only one study^27^used separate therapies and compared the groups. Additionally, all studies^20-22,24^included advice on parafunctional habits, diet, and pain management.

In our review, we found that there was a reduction in pain between the initial assessment and after the intervention, but no significant difference regarding whether the studies used the occlusal splints with exercises^20-22,24^or isolated therapies.^27^Only one study^24^evaluated the quality of life related to oral health (QLROH), observing an improvement in the dimensions of physical capacity and eating discomfort, regardless of the treatment used. Niemelä, et al.^22^(2012) and Katyayan, et al.^21^ (2013) evaluated mandibular movements and reported that the splints treatment was not more effective than exercises for increased mandibular mobility.

### Risk of bias

**Figure 4:**
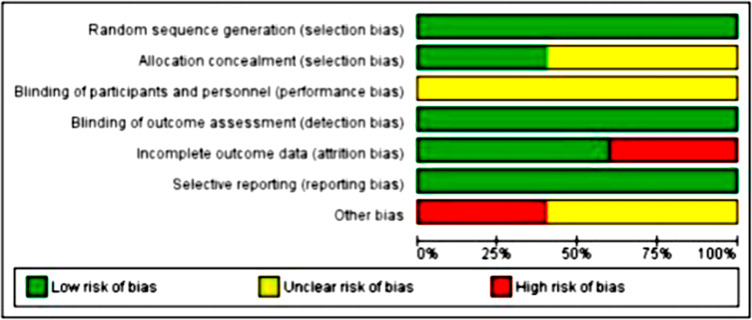
Risk of bias assessed by the Cochrane risk of bias tool

**Figure 5:**
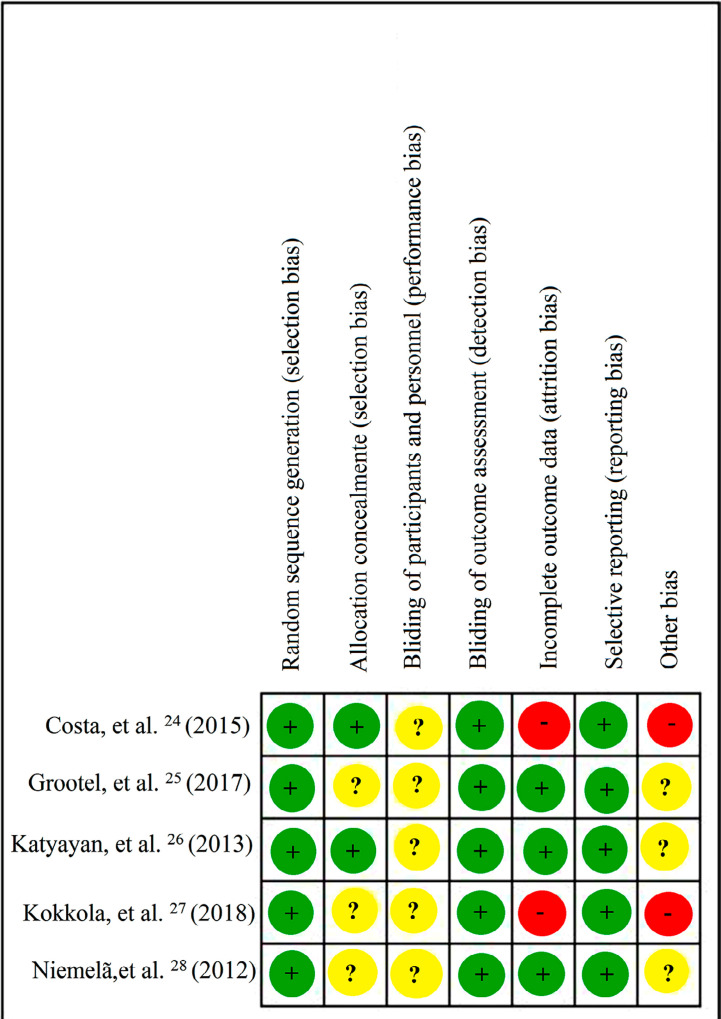
Risk of bias assessed by the Cochrane risk of bias tool

All included studies were classified as low risk of bias according to the Cochrane risk of bias tool,^27^although none of them met all the criteria for methodological quality since some information was unclear. Figure 4 shows a graph on the risk of bias and Figure 5 shows a more detailed analysis of each study.

## Discussion

This study aimed to verify whether exercises alone or associated with occlusal splints are effective in reducing pain in patients with TMD of myogenic origin. Therefore, RDC/TMD was used as the inclusion criterion, since it is considered the gold standard for diagnosis of TMJ dysfunction and is the protocol used over the diagnosis.^27,28^ The OS used in studies^22-26^ was made from heat-cured acrylic. According to Conti, et al.^12^ (2012) the occlusal splints promotes central, peripheral, and behavioral changes, reduces muscle activity, and improves occlusal stability. Melo, et al.^16^ (2020) also states that OS restores neuromuscular balance by promoting balanced condylar contact of the TMJ.

Regarding the protocol of splints use, patients were instructed to use it overnight, except for one study that advised that the splints should be used as long as possible or for at least 10-12 hours at night.^29^Deregibus, et al.^3^ (2021), in their randomized clinical trial (RCT), guided participants to use it at night, for at least 8 hours, while Melo, et al.^16^ (2020) stated that OS can be used in the morning or at night, depending on the patient’s clinical condition, which must be analyzed by a specialist. We should note that the Finnish guidelines recommend that splints should be used at night because it is uncomfortable to use during the day owing to work activities, school, and feeding.^30^However, the literature lacks consensus about the optimal period and time of occlusal splints usage.

Regarding the exercises used, Kokkola, et al.^26^ (2018), Niemelä, et al.^22^ (2012) and Katyayan, et al.^23^ (2013) implemented the protocol proposed by Carlsson and Magnusson^30^ (1999). This program consists of mouth opening, lateralization, and protrusion, actively and with maximum maintenance of the positions for a few seconds; it is, essentially, exercises that incorporate resistance from the patient’s fingers. These exercises are repeated from seven to ten times and performed two to three times per day. Costa, et al.^24^ (2015) used stretching and elongation exercises three to five times per second. Already Van Grootel, et al.^25^ (2017) performed the Jacobson method, which promoted the relaxation of the mandible muscles in addition to stretching, mandibular movement, and self-massage.

Fernández-de-las-Peñas and Piekartz^31^ (2020) state that mandibular exercises should be individualized aiming to improve muscle coordination, increase the range of motion, and relax strained muscles. There is no standardization of the exercises used to treat TMD, as to patient compliance with therapy, appropriate dosage, and exercise frequency. Despite this, it is possible to compare studies because of commonalities regarding relaxation, active movements, and stretching.

A common point in all studies^22-26^was the use of guidelines, advice, and instructions on the etiology and prognosis of the disease, diet, and parafunctional habits to avoid. Costa, et al.^24^ (2015) stressed the importance of sleep hygiene and the application of hot cushions on painful muscles. They also stated that counseling and behavioral changes were effective in relieving headaches attributed to TMD because the use of the occlusal splints did not offer any additional therapeutic effect. This reinforces the need for a more comprehensive protocol, involving professional intervention as well as counseling strategies and behavioral changes since these approaches are reported to be a valuable tool for the management of myofascial pain.^12^ Studies observed that patients with masticatory muscular pain have alterations in respiratory patterns, depression, and sleep disorders. Therefore, interventions that address bio-behavioral strategies, alone or in combination with other therapies, could help alleviate pain.^31,32^

The studies combining OS and exercises, making a comparison with only exercises,^22-24,26^ as well as the study comparing individual therapies, that is, only the OS compared with exercises,^25^ concluded that the treatments were effective in improving pain. However, no significant difference was observed between the treatment group and control groups.^22-26^We should highlight that OS and exercise therapy have similar success rates,^18^ with their efficacy being confirmed through follow-ups.^28^ Therefore, according to Van Grootel, et al.^25^ (2017) the key to the choice of treatment is patient compliance and preference.

The impact of pain on the quality of life in patients with TMD was assessed only by Kokkola, et al.^26^ (2018), who found that although the patients experienced an improvement in physical pain and discomfort when eating, no difference was observed between the interventions (OS and exercises vs exercises). However, TMD-related pain has a greater impact on QLROH than dental problems, and it is also known that patients with TMD suffer more, especially regarding the physical pain of TMD.^33,34^

Mandibular movements were evaluated by Niemelä, et al.^22^ (2012) who observed improvement after one month of treatment. However, there was no difference between the interventions performed (exercises + orientation vs OS + exercises + orientation). The improvement in movements was achieved in both groups and cannot be attributed only to the OS. Therefore, the improvement was more likely achieved because of the information received and the positive effects of both treatment methods used. The author also reported an adverse effect—increased pain upon palpation—in the splints group.

Katyayan, et al.^23^ (2013) also observed an improvement in mandibular movements between the beginning and after six months of treatment, but no significant difference between the groups. The author also emphasizes the need for further studies with sufficient sample sizes to assess the effectiveness of treatment with an occlusal splints on symptoms in TMD of myogenic origin.

Some methodological limitations in the included studies are that the samples were patients who intentionally sought treatment or who had been referred from orofacial pain centers. Additionally, the studies did not use the same treatment protocol for both exercise and splints use. Because of the heterogeneous nature of the data, which made quantitative synthesis difficult, performing a meta-analysis was impossible.

## Conclusion

The analyzed studies showed no difference in the improvement of pain, quality of life, and mandibular movements between the groups that performed only exercises or the associated treatments. Due to the heterogeneity of the studies analyzed, further research is needed.
